# Trismus, health-related quality of life, and trismus-related symptoms up to 5 years post-radiotherapy for head and neck cancer treated between 2007 and 2012

**DOI:** 10.1007/s00520-023-07605-w

**Published:** 2023-02-14

**Authors:** Susan Aghajanzadeh, Therese Karlsson, Lisa Tuomi, My Engström, Caterina Finizia

**Affiliations:** 1 Department of Otorhinolaryngology, Head and Neck Surgery, Institute of Clinical Sciences, Sahlgrenska Academy, University of Gothenburg, Gothenburg, Sweden; 2 Department of Otorhinolaryngology, Head and Neck Surgery, Sahlgrenska University Hospital, Region Västra Götaland, Gothenburg, Sweden; 3 Institute of Neuroscience and Physiology, Speech and Language Pathology Unit, Sahlgrenska Academy, University of Gothenburg, Gothenburg, Sweden; 4 Institute of Health and Care Sciences, Sahlgrenska Academy, University of Gothenburg, Gothenburg, Sweden; 5 Department of Surgery, Sahlgrenska University Hospital, Region Västra Götaland, Gothenburg, Sweden

**Keywords:** Trismus, Radiotherapy, Health-related quality of life, Mouth opening ability, Head and neck cancer

## Abstract

**Purpose:**

Radiotherapy-induced trismus is present in up to 40% of patients treated radiotherapeutically for head and neck cancer (HNC) and impacts health-related quality of life (HRQL) negatively. This prospective study aimed to investigate the development of trismus and its influence on HRQL and trismus-related symptoms in HNC patients for up to 5 years post-radiotherapy completion as no such follow-up studies exist.

**Methods:**

Patients (*n* = 211) were followed prospectively from pre-radiotherapy to 12 and 60 months post-radiotherapy. At each follow-up, maximum interincisal opening (MIO) was measured, and patients filled in the European Organization for Treatment of Cancer Quality-of-Life Questionnaire Core-30 (EORTC QLQ-C30), Head and Neck-35 (EORTC QLQ-HN35), and Gothenburg Trismus Questionnaire (GTQ). Trismus was defined as an MIO ≤ 35 mm.

**Results:**

At 1 year post-radiotherapy, a total of 27% met the trismus criterion, and at 5 years post-radiotherapy, the corresponding figure was 28%. Patients in the trismus group scored significantly worse compared to the patients without trismus on 8/15 domains at 1 year post-radiotherapy on EORTC QLQ-C30, further worsening in 11/15 domains at 5 years post-radiotherapy. Similar results were found for EORTC QLQ-HN35. Patients with trismus reported more trismus-related symptoms according to the GTQ at both timepoints compared to those without trismus.

**Conclusion:**

This study highlights that HNC patients suffering from radiotherapy-induced trismus report poorer HRQL and more trismus-specific symptoms compared to patients without trismus. These differences persist and increase up to at least 5 years following treatment completion. Hence, our results highlight that radiotherapy-induced trismus affects long-term HRQL, jaw symptoms, and pain, further stressing the need for early and structured intervention.

## Introduction

Over the past 30 years, the epidemiology of head and neck cancer (HNC) has changed, with a decline seen in tobacco- and alcohol-related HNC and an increase in human papillomavirus (HPV)-related HNC. In Sweden, there has been a steady increase in the annual incidence of HNC with a 38% increase in new cases reported by the National Quality Registries in Swedish Health Care between 2008 and 2019 [1].

Tumors of HNC are most commonly treated surgically or radiotherapeutically with or without the addition of chemotherapy [2]. In general, patients with HPV-related HNC are younger and have a better overall survival prognosis than those with other HNC [3, 4]. As the survival rates increase for patients treated for HNC, an increased interest among clinicians and researchers has developed to optimize long-term function and improve health-related quality of life (HRQL) among these affected individuals. One burdensome, potentially long-term complication that may occur in up to 40% of patients after oncologic treatment of HNC is trismus, or restricted mouth opening [5–7]. The most widely used criterion for trismus is that of Dijkstra et al. [8], which defines trismus as a maximum interincisal opening (MIO) ≤ 35 mm.

Previous studies focusing on post-radiotherapy (RT) trismus have demonstrated that trismus impacts HRQL negatively and may cause pain as well as interference with normal daily activities such as speaking or eating [9–12]. As no follow-up studies regarding HRQL beyond 3 years post-RT completion exist, it is yet unclear whether these negative effects persist, which exposes an important knowledge gap in the literature.

Hence, this prospective observational cohort study aims to investigate the development of trismus and its influence on HRQL and trismus-related symptoms, comparing HNC patients who develop postradiation trismus to those who do not for up to 5 years post-RT.

## Materials and methods

### Study participants and design

Patients were invited to participate in this study in connection with the weekly multidisciplinary tumor board meetings for all new cases of diagnosed HNC in the region of West Sweden at Sahlgrenska University Hospital in Gothenburg, between 2007 and 2012. The Sahlgrenska University Hospital is one of the major university hospitals in Northern Europe and the specialized center for HNC treatment for the Swedish county of Vastra Gotaland, populated by 1.7 million. Inclusion criteria were 18 years of age or older, receiving curatively intended RT, and cancer sites in the oral cavity, oropharynx, nasopharynx, salivary glands, paranasal sinuses, or cervical carcinoma of unknown primary (CCUP). Exclusion criteria included pre-treatment MIO of ≤ 35 mm, recurrent disease, surgical treatment alone, and insufficient cognitive abilities and Swedish language competency to independently complete the questionnaires and partake in examinations. Patients were followed prior to RT (baseline) and at 12 and 60 months post-RT. At each follow-up, MIO was measured, and the patients filled in patient-reported outcome instruments related to HRQL and trismus-specific symptoms, as described below. The Adult Comorbidity Index 27 (ACE27) and Karnofsky Performance Status Scale were filled in at baseline only [13, 14].

### Oncologic treatment

Tumors were classified according to the global standard, the TNM-staging system by the International Union Against Cancer (UICC), which includes tumor size (T), regional lymph nodes involvement (N), and distant metastasis (M) [15]. Curatively intended RT was administered according to regional guidelines. During 2007–2009, external RT was generally administered through accelerated hyperfractionated therapy as 1.7 Gray (Gy) per fraction 5 days per week, totaling 64.6 Gy. During 2010–2012, the external RT was administered through accelerated fractioning as 2 Gy per fraction one or two times per day for 5 days per week with a total of six treatments per week, totaling 68 Gy. Induction (cisplatin and 5-fluoruracil) or concomitant (weekly cisplatin) chemotherapy was added to the RT for patients with stage III to IV disease in accordance with local treatment standards (*n* = 141).

Surgery was performed with post-operative RT with or without the addition of chemotherapy for some tumor sites. Oral tumors stage III–IV were treated with surgery, including diagnostic and therapeutic neck dissections and radiotherapy. If the tumor was assessed to be unresectable, the patient received chemoradiotherapy only.

Furthermore, primary site tumors were removed surgically, including neck dissection, followed by post-operative RT also if located in the salivary glands and sinonasal cancers. Neck dissections were also performed before administering non-surgical oncologic treatment in selected cases of those diagnosed with CCUP, depending on the status of their nodal metastasis. Non-surgical treatment was clinical practice for oropharyngeal and nasopharyngeal tumors.

### Outcome measures

#### Maximum interincisal opening (MIO)

MIO was measured in millimeters with a ruler as the maximum distance between the upper and lower incisors at all three timepoints. The cut-off point defining trismus was an MIO of ≤ 35 mm [8]. Edentulous patients measured MIO with the dental prosthesis in place.

#### The European Organization for Research and Treatment of Cancer

The European Organization for Research and Treatment of Cancer Quality-of-Life Questionnaires (EORTC QLQ) were used to measure HRQL at all timepoints. Firstly, the EORTC QLQ Core-30 (QLQ-C30), which is a cancer-specific instrument, was utilized [16]. In addition to this, the complementary head and neck disease-specific module EORTC QLQ-HN35 was added for depth regarding HNC-specific symptoms [17].

The EORTC QLQ-C30 consists of five function scales, a global quality of life scale, three symptom scales, and six single items, totaling 30 items that describe the patients’ symptoms and functional level during the last week. The EORTC QLQ-HN35 consists of 35 items addressing symptoms specific to HNC and its treatments. Scores range from 0 to 100, where higher scores represent better function in the functioning domains and a higher global quality of life, whereas higher scores in the symptom domains equate to a higher symptom burden [18].

#### Gothenburg Trismus Questionnaire (GTQ)

The GTQ is the first comprehensive trismus-specific questionnaire and was developed as a complement to the objective measure of MIO. The GTQ is composed of three domains containing 13 items: jaw-related problems (six items), eating limitations (four items), and muscular tension (three items). Additionally, there are eight single items. The scores of the domains and single items range from 0 to 100, where a higher score indicates more symptoms and lower scores represent less symptoms [19].

### Statistical analysis

For categorical variables, number and percentages are presented. Standard deviation is presented for relevant variables related to sociodemographic data in Table 1. Continuous variables are presented using mean and 95% confidence interval. For comparisons between groups, Fischer’s exact test was used for dichotomous variables, Mantel-Haenszel chi-square test for ordered categorical variables, Mann–Whitney *U* test for continuous variables, and chi-square test for non-ordered categorical variables. All tests were two-sided and conducted at a 5% significance level. SAS version 9.4 was used for analyses.

**Table 1:** Sociodemographic data at baseline and 5-year follow-up (mean, *n*, SD, 95% CI, %)

	Baseline (*n* = 211)	5 years post-RT (*n* = 129)		*P* value ^a^
		Trismus (*n* = 36)	No trismus (*n* = 93)	
	Mean (range; SD) 95% CI	Mean (range; SD) 95% CI	Mean (range; SD) 95% CI	
Age	61.0 (29–86; 10.2) 60–62	59.3 (29–80; 9.6) 59–61	59.6 (31–80; 9.8) 59–62	0.88
Karnofsky Performance Scale ^b^	97.5 (60–100; 6.6) 97–98	98.3 (70–100; 6.1) 97–99	98.8 (70–100; 4.6) 98–99	0.86
	*n* (%)	*n* (%)	*n* (%)	
Sex				
Male	157 (74)	26 (72)	66 (71)	1.00
Female	54 (26)	10 (28)	27 (29)	
Living alone				
Yes	58 (27)	12 (33)	18 (19)	0.18
No	153 (73)	24 (67)	75 (81)	
Educational years				
6-9 years	58 (27)	6 (17)	24 (26)	0.69
9-12 years	99 (47)	20 (55)	40 (43)	
>12 years	54 (26)	10 (28)	29 (31)	
Employment status				
Full time	98 (46)	19 (53)	49 (53)	0.96
Part time	16 (8)	3 (8)	8 (8)	
Unemployed	8 (4)	1 (3)	2 (2)	
Old age pensioner	70 (33)	8 (22)	25 (27)	
Early retiree	19 (9)	5 (14)	9 (10)	
Dental status				
Upper prosthesis	2 (1)	0 (0)	0 (0)	0.87
Lower prosthesis	1 (1)	0 (0)	1 (1)	
Both upper+lower prosthesis	4 (2)	1 (3)	0 (0)	
Own teeth	204 (96)	35 (97)	92 (99)	
Smoking				
Yes	49 (23)	28 (78)	72 (77)	1.00
No	162 (77)	8 (22)	21 (23)	
BMI mean (range; SD), 95% CI	26.7 (17–46; 4.4) 26–27	27.3 (22–40; 4.1) 27-28	26.5 (17–37; 3.7) 26–28	0.80
BMI group				
< 18.5	3 (2)	0 (0)	2 (2)	1.00
18.5–25	68 (32)	10 (28)	27 (29)	
> 25	139 (66)	26 (72)	64 (69)	
Missing = 1				
Cancer stage				
I	8 (4)	2 (6)	9 (10)	0.69
II	36 (17)	7 (19)	22 (23)	
III	49 (23)	10 (28)	14 (15)	
IV	118 (56)	17 (47)	48 (52)	
Tumor site				
Oral cavity	34 (16)	7 (19)	8 (9)	0.14
Oropharynx	132 (62)	19 (53)	65 (69)	
Salivary gland	8 (4)	1 (3)	4 (4)	
Nasopharynx/sinuses	16 (8)	2 (6)	8 (9)	
CCUP	21 (10)	7 (19)	8 (9)	
Cancer treatment				
RT only	45 (21)	6 (17)	15 (16)	0.07
Surgery + [C]RT	35 (17)	13 (36)	15 (16)	
CRT	131 (62)	17 (47)	63 (68)	
ACE27				
No comorbidity	92 (44)	21 (58)	43 (46)	0.89
Mild comorbidity	76 (36)	8 (22)	36 (39)	
Moderate comorbidity	35 (16)	5 (14)	14 (15)	
Severe comorbidity	8 (4)	2 (6)	0 (0)	

Abbreviations: *ACE27* Adult Comorbidity Evaluation 27, *BMI* body mass index, *CCUP* cervical carcinoma of unknown primary, *CRT* chemoradiotherapy, *RT* radiotherapy, *SD* standard deviation. ^a^*P* values comparing data between the trismus versus no trismus groups. ^b^Range from 0 to 100 equals perfect health

### Ethical considerations

The study was conducted according to the Declaration of Helsinki and was approved by the Regional Ethic Review Board in Gothenburg, Sweden. All participants gave their written informed consent before study inclusion.

## Results

### Sociodemographic and clinical data

A total of 211 patients were included. The baseline sociodemographic and clinical characteristics can be found in Table 1. At the 12-month follow-up, 180 (85%) patients remained enrolled in the study and at the 5-year follow-up the corresponding number was 129 (61%). Reasons for drop-out at 5 years post-RT were tumor recurrence (*n*=39), poor general health (*n*=4), death due to unknown cause (*n*=13) and unknown (*n*=26). The 129 patients completing the study had at baseline significantly higher Karnofsky scores, i.e. better performance status (98.7 vs 95.6, *p*<0.001), less comorbidity as measured by ACE27 (*p*=0.006) and were younger (59.5 vs 63.4 years, *p*=0.009) compared to the 82 patients who dropped out.

### MIO

At baseline, as part of the inclusion criteria, no patients suffered from trismus (*n* = 211). At 1 year post-RT, however, a total of 27% (*n* = 49) met the trismus criterion, and at 5 years after finishing RT, the corresponding figure was 28% (*n* = 36, Table 2).

**Table 2:** MIO at pre-radiotherapy, 1 and 5 years post-radiotherapy for patients with and without trismus (mean (95% CI))

	Pre-RT	1 year post-RT			5 years post-RT		
	No trismus (*n* = 211)	Trismus (*n* = 49)	No trismus (*n* = 131)	*P* value	Trismus (*n* = 36)	No trismus (*n* = 93)	*P* value
	Mean (95% CI)	Mean (95% CI)	Mean (95% CI)		Mean (95% CI)	Mean (95% CI)	
MIO	51.5 (51–52)	31.3 (30–33)	45.5 (44–47)	< 0.001	30.4 (29–32)	45.5 (44–47)	< 0.001

Abbreviations: *MIO* maximum interincisal opening, *RT* radiotherapy, *CI* confidence interval

### EORTC QLQ C30 and HN35

Results from the EORTC QLQ-C30 are presented in Table 3. The patients in the trismus group scored significantly worse compared to the patients with no trismus on eight of 15 domains at 1 year post-RT. The domains in question were physical, role, and social function, as well as global quality of life, fatigue, pain, appetite loss, and financial loss. At 5 years post-RT, patients suffering from trismus reported significantly worse scores in 11 out of 15 domains. This included most functional and all symptom items, as well as the single items dyspnea, appetite loss, and financial loss.

**Table 3:** EORTC QLQ C30 at pre-radiotherapy, 1 and 5 years post-radiotherapy for patients with and without trismus (mean (95% CI))

	Pre-RT	1 year post-RT			5 years post-RT		
	No trismus (*n* = 211)	Trismus (*n* = 49)	No trismus (*n* = 131)	*P* value	Trismus (*n* = 36)	No trismus (*n* = 93)	*P* value
	Mean (95% CI)	Mean (95% CI)	Mean (95% CI)		Mean (95% CI)	Mean (95% CI)	
Functioning domains							
Physical function	89.7 (87–92)	81.9 (77–87)	88.5 (86–91)	0.03	81.4 (74–89)	84.9 (81–89)	0.42
Role function	74.2 (70–79)	65.6 (56–75)	82.5 (78–87)	< 0.001	70.6 (59–82)	85.9 (81–91)	0.01
Emotional function	70.1 (67–73)	76.2 (69–84)	83.6 (80–87)	0.06	76.0 (67–85)	87.1 (83–91)	< 0.01
Cognitive function	85.0 (83–88)	81.3 (75–88)	86.4 (83–90)	0.14	74.5 (65–84)	87.1 (84–90)	< 0.01
Social function	82.9 (80–86)	71.8 (63–81)	84.0 (80–88)	< 0.01	73.0 (63–83)	87.4 (83–92)	< 0.01
Global quality of life	65.8 (63–69)	62.8 (56–70)	71.3 (68–75)	0.02	61.0 (51–71)	75.5 (71–80)	< 0.01
Symptom scales							
Fatigue	23.0 (20–26)	32.2 (25–40)	23.8 (20–28)	0.04	36.6 (23–50)	20.5 (16–26)	< 0.01
Nausea/vomiting	4.6 (3–6)	6.8 (3–11)	3.6 (2–6)	0.14	8.8 (2–16)	2.5 (1–5)	0.03
Pain	23.7 (20–27)	24.1 (16–32)	15.2 (12–19)	0.03	31.4 (19–43)	12.3 (8–17)	< 0.001
Single items							
Dyspnoea	15.5 (12–19)	22.4 (15–30)	15.6 (12–19)	0.08	32.3 (22–43)	15.8 (11–20)	< 0.01
Insomnia	27.9 (24–32)	21.2 (12–30)	18.7 (14–24)	0.61	27.5 (16–39)	20.9 (15–27)	0.27
Appetite loss	12.9 (10–16)	31.3 (21–42)	14.9 (10–20)	< 0.01	17.6 (9–26)	6.5 (3–10)	0.01
Constipation	8.5 (6–11)	10.9 (5–17)	9.52 (6–13)	0.68	14.7 (5–25)	8.7 (5–12)	0.17
Diarrhoea	4.3 (3–6)	5.44 (1–10)	5.11 (2–8)	0.87	6.9 (2–15)	5.2 (2–8)	0.61
Financial loss	13.5 (10–17)	24.5 (15–34)	14.0 (9–19)	0.03	17.6 (7–28)	7.0 (3–11)	0.03

Abbreviations: *RT* radiotherapy, *CI* confidence interval

Scores range from 0 to 100. Higher scores in the function domains and global quality of life indicate better function, whereas higher scores in symptom domains indicate increased symptom burden

Patients with trismus also reported worse symptoms compared to those without trismus in the EORTC QLQ HN35-variables speech, teeth, and mouth opening at 1 year post-treatment completion. The same variables also appeared significantly worse in the trismus group at the 5-year follow-up in addition to other variables, including pain, senses, social eating, dry mouth, sticky saliva, and feeling ill. The items where both groups reported the highest symptom burden at both follow-up timepoints were dry mouth and sticky saliva. The results from EORTC QLQ-HN35 are presented in Table 4.

**Table 4:** EORTC QLQ HN35 at pre-radiotherapy, 1 and 5 years post-radiotherapy for patients with and without trismus (mean (95% CI))

	Pre-RT	1 year post-RT			5 years post-RT		
	No trismus (*n* = 211)	Trismus (*n* = 49)	No trismus (*n* = 131)	*P* value	Trismus (*n* = 36)	No trismus (*n* = 93)	*P* value
	Mean (95% CI)	Mean (95% CI)	Mean (95% CI)		Mean (95% CI)	Mean (95% CI)	
Symptom domains							
Pain	21.9 (19–25)	26.0 (19–33)	21.4 (18–25)	0.21	28.3 (19–38)	13.0 (9–17)	< 0.001
Swallowing	14.4 (11–17)	25.5 (19–32)	19.4 (15–23)	0.12	25.0 (15–36)	16.2 (12–21)	0.08
Senses	9.1 (7–12)	27.9 (21–35)	30.3 (25–35)	0.61	31.0 (22–40)	18.2 (13–23)	0.02
Speech	12.3 (10–15)	22.3 (16–28)	12.6 (10–16)	< 0.01	23.2 (14–32)	10.4 (7–14)	< 0.01
Social eating	13.2 (10–16)	34.0 (27–42)	23.8 (19–30)	0.05	27.5 (18–37)	14.2 (10–19)	< 0.01
Social contact	7.1 (5–10)	12.9 (8–18)	7.3 (4–10)	0.06	14.1 (6–22)	7.1 (4–10)	0.06
Sexuality	28.3 (23–33)	38.5 (28–49)	28.8 (23–35)	0.10	33.3 (22–45)	32.9 (25–41)	0.95
Symptom single items							
Teeth	7.5 (5–10)	27.2 (17–37)	16.8 (12–21)	0.04	34.3 (22–47)	17.8 (11–24)	0.02
Open mouth	8.7 (6–12)	42.2 (33–51)	15.5 (12–19)	< 0.001	47.6 (36–60)	12.5 (8–17)	< 0.001
Dry mouth	18.1 (14–22)	72.8 (64–81)	62.9 (57–69)	0.06	66.7 (56–78)	51.6 (45–59)	0.02
Sticky saliva	16.5 (13–20)	53.5 (44–63)	54.2 (48–61)	0.90	62.7 (43–82)	38.8 (31–46)	< 0.01
Cough	16.9 (14–20)	19.0 (12–26)	21.6 (17–27)	0.58	30.5 (19–42)	19.8 (14–25)	0.07
Felt ill	18.3 (15–22)	19.4 (12–27)	14.1 (10–19)	0.22	22.9 (13–33)	7.7 (4–11)	< 0.01
Nutritional supplement	12.5 (8–17)	32.7 (19–46)	18.9 (12–26)	0.06	25.7 (11–41)	13.2 (6–20)	0.10

Abbreviations: *RT* radiotherapy, *CI* confidence interval

Scores range from 0 to 100. Higher scores in symptom domains indicate increased symptom burden

### GTQ

The patients in the trismus group reported significantly more symptoms in all three GTQ domains (jaw-related problems, eating limitations, and muscular tension) at 5 years post-RT. At 1 year after RT completion, every symptom except for experiencing facial pain right now was statistically significantly worse in the trismus group. The GTQ results are presented in Table 5.

**Table 5:** GTQ at pre-radiotherapy, 1 and 5 years post-radiotherapy for patients with and without trismus (mean (95% CI))

	Pre-RT	1 year post-RT			5 years post-RT		
	No trismus (*n* = 211)	Trismus (*n* = 49)	No trismus (*n* = 131)	*P* value	Trismus (*n* = 36)	No trismus (*n* = 96)	*P* value
	Mean (95% CI)	Mean (95% CI)	Mean (95% CI)		Mean (95% CI)	Mean (95% CI)	
Domains							
Jaw-related problems	9.6 (7–12)	38.9 (33–45)	18.8 (15–23)	< 0.001	46.0 (37–55)	13.1 (9–17)	< 0.001
Eating limitations	11.7 (8–15)	36.7 (30–44)	22.3 (18–27)	< 0.001	41.4 (33–50)	18.5 (13–24)	< 0.001
Muscular tension	7.0 (5–9)	28.7 (23–35)	13.5 (11–16)	< 0.001	36.9 (31–43)	17.8 (13–23)	< 0.001
Single items							
Facial pain							
Right now	9.2 (7–12)	16.7 (11–22)	11.0 (8–14)	0.07	23.8 (16–32)	7.1 (4–10)	< 0.001
Pain when worst last month	22.3 (19–26)	27.2 (20–34)	16.5 (13–20)	< 0.01	28.6 (19–38)	9.2 (6–13)	< 0.001
Pain average	18.7 (16–22)	26.9 (20–34)	15.0 (12–19)	< 0.01	28.6 (20–37)	7.7 (5–11)	< 0.001
Pain interfering with social, leisure, and family activities	6.4 (4–9)	15.1 (9–22)	5.8 (3–8)	< 0.01	27.1 (16–38)	4.1 (1–7)	< 0.001
Pain affecting ability to work last month	9.4 (6–13)	16.8 (9–25)	6.6 (3–10)	< 0.01	23.6 (14–33)	2.8 (1–5)	< 0.001
LOM	8.3 (6–11)	42.7 (36–50)	17.5 (13–22)	< 0.001	42.9 (34–52)	14.3 (10–19)	< 0.001
LOM interfering with social, leisure, and family activities	4.1 (2–6)	22.9 (15–30)	6.0 (4–9)	< 0.001	25.7 (16–35)	5.6 (2–9)	< 0.001
LOM affecting ability to work	4.4 (2–7)	20.3 (13–28)	6.9 (4–10)	< 0.001	24.3 (14–34)	3.3 (1–6)	< 0.001

Abbreviations: *LOM* limitations in mouth opening, *RT* radiotherapy, *CI* confidence interval. The scores of the domains and single items range from 0 to 100, where a higher score indicates more symptoms

## Discussion

This study is, to the best of our knowledge, the longest follow-up study describing HRQL in HNC patients with post-RT trismus. Its large prospective cohort of patients and longitudinal design has yielded important findings in terms of HRQL prevalence and trismus-related symptoms.

Firstly, the trend that patients suffering from trismus report generally worse HRQL scores compared to those without trismus is in line with previous findings of shorter-term studies, but highlights that these deteriorations persist long term [10, 20, 21]. The longer follow-up period in this study further revealed interesting findings as the disadvantageous scoring persisted at 5 years post-RT. In addition, more HRQL and pain domains displayed statistically significant deteriorations compared to at 12 months post-RT. The prevalence of trismus remained fairly constant at 27–28% between the 12-month and 5-year follow-up and, hence, cannot explain the continued deterioration reported in HRQL. The endpoint deterioration seen in the EORTC QLQ HN35 domains, senses, sticky saliva, and dry mouth, could, however, be attributed to the development of late radiation-induced effects [22]. Epstein et al. [23] concluded that both fraction size, treatment time, and radiated tissue volume impacted the development of mucositis, and Schultz et al. [24] found a 78% incidence of hyposalivation in their 88 patients, where higher radiation doses increased the risk of xerostomia four-fold. Hence, other factors influenced by tumor site, treatment type, and dosage of RT also impact HRQL.

Other aspects known to contribute to HRQL might be found in the patient characteristics. Patients who live alone have been found more likely to report a negative impact on quality of life [25, 26]. Even though there was no statistically significant difference in the present study at 5 years post-RT, a total of 33% of the participants with trismus reported living alone, whereas the corresponding number among the participants without trismus was 19%. This could be a contributing factor to the differences found in the domains social function and global quality of life. In a survey study by Dahill et al. [27], loneliness after HNC treatment was associated with a worse overall quality of life. The study was however limited by a low response rate and insufficient information about for example marital status and living alone or not. Research regarding patients with HNC and partner/spousal support or methods for coping is still modestly conducted. A cross-sectional study on cancer patients by Sayilan et al. showed married individuals to have better social support levels than single individuals [28]. Additionally, the presence of comorbidities and a higher tumor stage have been associated with worse HRQL, where a somewhat larger, although not statistically significant, extent of the participants with trismus had more advanced disease and more comorbidities [26].

Additional important findings in this material highlighted that the trismus group reported worse scores with regard to all pain modules in the EORTC QLQ-HN35 and the GTQ compared to those without trismus. Scores continued to deteriorate over time for patients with trismus, whereas on the other hand, the quantitative scores for patients without trismus improved at the 5-year follow-up. Thus, a statistically significant pattern regarding pain emerges over time, which appears disadvantageous for those suffering from trismus. These results are in line with a previous study regarding pain and its association with trismus, where structured exercise aiming to improve jaw-opening also resulted in reduced pain 3 years following oncologic treatment. This indicates that trismus is a prominent reason for post-RT pain [12]. Interestingly, the majority of domains and items in both the EORTC QLQ-C30 and QLQ-HN35 improved to match or surpass scores reported at baseline for patients without trismus at the 5-year follow-up.

Overall, the findings in this study highlight and further confirm the presence and persistence of long-term trismus, trismus-specific symptoms, and deteriorations in HRQL for those suffering from the condition. Previous research has demonstrated that patients who perform structured jaw exercises also report less trismus and better scoring regarding HRQL. A prospective interventional study by Karlsson et al. reported on 100 HNC patients with trismus of which 50 underwent a 10-week structured exercise program and the remaining constituted a control group. Patients were followed up to 3 years post-RT, and at all study timepoints, MIO was higher for the intervention group along with better reported HRQL scores [29]. Furthermore, a separate study by Pauli et al. concluded that HNC patients who experienced jaw-related pain prior to oncologic treatment were at higher risk of developing radiation-induced trismus after 6 months compared to those without jaw-related pain, thus highlighting the important of optimizing patients’ pain treatment and oral health even before starting radiation treatment [30].

### Clinical implications

As it is now known that trismus is a long-term complication severely affecting HRQL and that structured jaw training improves MIO, the importance of routinely introducing rehabilitative measures should continue to be emphasized. However, more recent studies have also focused on prophylactic intervention. A recent systematic review by Wang et al. covering prehabilitative interventions for trismus prevention identified a total of 11 studies, of which six contained sufficient information for analysis [31]. Results are promising both in terms of MIO and HRQL, yet all studies were severely hampered by small study sizes, short-term follow-up of a maximum of 2 years as well as a varying range of implemented intervention modalities, often lacking an objective MIO-measure and thereby resulting in low confidence ratings. Thus, studies investigating prehabilitative trismus strategies long-term should also be conducted.

### Strengths and limitations

The strength of this study lies in its prospective, longitudinal design and in the size of the cohort. Furthermore, objective measurements have been used for MIO, and patient-reported outcome data have been obtained through validated, reliable, and widely used HRQL instruments. The study is limited by the patients who dropped out as these were in slightly worse overall health at baseline, raising the awareness of survival bias in long-term studies. Another further possible limitation includes the period that the data was collected. Since then, treatment methods and regimens have somewhat changed which may lead to an altered prevalence of post-RT trismus and hence associated HRQL data.

## Conclusion

This study highlights that HNC patients suffering from post-RT trismus report worse HRQL and trismus-specific symptoms compared to patients not suffering from post-RT trismus. These differences not only persist but increase up to at least 5 years following treatment completion. The results from the study highlight that RT-induced trismus affects long-term HRQL, jaw symptoms, and pain, further stressing the need for intervention in this patient group.
